# Effect of Broccoli Sprouts on Nasal Response to Live Attenuated Influenza Virus in Smokers: A Randomized, Double-Blind Study

**DOI:** 10.1371/journal.pone.0098671

**Published:** 2014-06-09

**Authors:** Terry L. Noah, Hongtao Zhang, Haibo Zhou, Ellen Glista-Baker, Loretta Müller, Rebecca N. Bauer, Megan Meyer, Paula C. Murphy, Shannon Jones, Blanche Letang, Carole Robinette, Ilona Jaspers

**Affiliations:** 1 Center for Environmental Medicine, Asthma and Lung Biology, University of North Carolina at Chapel Hill, Chapel Hill, North Carolina, United States of America; 2 Department of Pediatrics, University of North Carolina at Chapel Hill, Chapel Hill, North Carolina, United States of America; 3 Department of Biostatistics, University of North Carolina at Chapel Hill, Chapel Hill, North Carolina, United States of America; 4 Curriculum in Toxicology, University of North Carolina at Chapel Hill, Chapel Hill, North Carolina, United States of America; 5 Department of Microbiology and Immunology, University of North Carolina at Chapel Hill, Chapel Hill, North Carolina, United States of America; 6 Biological and Biomedical Sciences Program, University of North Carolina at Chapel Hill, Chapel Hill, North Carolina, United States of America; 7 University Children's Hospital Basel, Basel, Switzerland; University of South Dakota, United States of America

## Abstract

**Background:**

Smokers have increased susceptibility and altered innate host defense responses to influenza virus infection. Broccoli sprouts are a source of the *Nrf2* activating agentsulforaphane, and short term ingestion of broccoli sprout homogenates (BSH) has been shown to reduce nasal inflammatory responses to oxidant pollutants.

**Objectives:**

Assess the effects of BSH on nasal cytokines, virus replication, and *Nrf2*-dependent enzyme expression in smokers and nonsmokers.

**Methods:**

We conducted a randomized, double-blind, placebo-controlled trial comparing the effects of BSH on serially sampled nasal lavage fluid (NLF) cytokines, viral sequence quantity, and *Nrf2*-dependent enzyme expression in NLF cells and biopsied epithelium. Healthy young adult smokers and nonsmokers ingested BSH or placebo (alfalfa sprout homogenate) for 4 days, designated Days -1, 0, 1, 2. On Day 0 they received standard vaccine dose of live attenuated influenza virus (LAIV) intranasally. Nasal lavage fluids and nasal biopsies were collected serially to assess response to LAIV.

**Results:**

In area under curve analyses, post-LAIV IL-6 responses (P = 0.03) and influenza sequences (P = 0.01) were significantly reduced in NLF from BSH-treated smokers, whileNAD(P)H: quinoneoxidoreductasein NLF cells was significantly increased. In nonsmokers, a similar trend for reduction in virus quantity with BSH did not reach statistical significance.

**Conclusions:**

In smokers, short term ingestion of broccoli sprout homogenates appears to significantly reduce some virus-induced markers of inflammation, as well as reducing virus quantity. Nutritional antioxidant interventions have promise as a safe, low-cost strategy for reducing influenza risk among smokers and other at risk populations.

**Trial Registration:**

ClinicalTrials.gov NCT01269723

## Introduction

Influenza virus infection is a serious, large-scale global public health problem. Current vaccines and antiviral drugs are only partially effective against influenza, and may be ineffective in the setting of epidemics due to antigenic shift or emergence of drug-resistant strains [1], [2]. Thus, novel strategies for controlling influenza are needed, especially for susceptible subpopulations such as those with chronic disease, or with chronic exposure to airborne pollutants. Tobacco smoke has been repeatedly shown to be a significant risk factor for severe influenza infection, whether from active smoking[3]–[8] or from second-hand exposures[9].

Using transient low-level nasal infection with standard dose of live attenuated influenza virus (LAIV) vaccine as a model, we have previously shown that healthy young adult smokers have blunted host defense responses to LAIV and enhanced viral replication, compared to healthy nonsmokers [10]. We have also shown that epithelial antiviral responses to influenza *in vivo* and in experimental cell culture models are blunted in smokers, and that the abnormalities can be corrected *in vitro*with antioxidants including sulforaphane(SFN) [11]–[13]. SFN, which has shown promise as a potent antioxidant and chemopreventive agent due to its upregulation of*Nrf2*-dependent, cytoprotective Phase II antioxidant enzymesincluding heme oxygenase-1 (HMOX1) and NAD(P)H: quinoneoxidoreductase (NQO1), is present in high concentrations in broccoli sprouts[14], [15]. Prior investigation in human volunteers has demonstrated a measurable,dose-dependent, short-term effect of broccoli-sprout homogenates on expression of *Nrf2*-dependent enzymes in the upper airway[16]and in the skin [17]. Additionally, broccoli sprouts were shown to reduce nasal allergic inflammation after diesel particle exposure[18].

We hypothesized that short term ingestion of broccoli sprouts would have a beneficial effect on nasal responses to influenza virus in smokers, by preventing the enhanced virus quantity and blunted IL-6 responses we observed in our previous study. Using LAIV as a model infection as previously reported[10], [19], we carried out an interventional study in otherwise-healthy smokers and in nonsmoking, healthy volunteers. Our results suggest that antioxidant-focused, “nutraceutical” approaches may be a promising strategy to pursue as a low-costadjunct to existingapproaches for reducing the impact of influenza on individuals exposed to airborne oxidant pollutants.

## Materials And Methods

### CONSORT checklist and IRB protocol

The protocol for this trial and supporting CONSORT checklist are available as supporting information; see Checklist S1 and Protocol S1.

### Study design and subjects

To confirm that a broccoli sprout homogenate (BSH) preparation would upregulate antioxidant enzymes in nasal cells similarly to the previously published study of Riedl et al. [16], we initially conducted a pilot study in 10 healthy young adult volunteers. We then carried out a randomized, double-blind, placebo-controlled study measuring the effect of short-term ingestion of BSH,compared to alfalfa sprout homogenate (ASH) controls, on nasal mucosal host defense response to a standard nasal vaccine dose of LAIV. Randomization was 1∶1 and the randomization method was by the permuted blocks method.

Subjects underwent screening for smoking history, informed consent, and randomization. A baseline superficial scrape biopsy of the epithelium was performed on the inferior surface of the middle nasal turbinate and epithelial RNA was isolated. Three to four weeks later, one BSH or alfalfa sprout control (ASH) shake was ingested daily for 4 consecutive days, designated days −1, 0, 1, and 2 (see belowfor preparation method). Shakes were ingested by subjects under direct observation by study staff. On day 0, a standard vaccine dose of LAIV (MedImmune, Inc.) was administered into each nostril as a nasal spray according to the manufacturer's recommendations. Nasal lavage was done on days −1, 0, 1, 2, 3, and 7. Anothersuperficial nasal biopsy on the inferior surface of the middle turbinate was done on day 2 and processed for qRT-PCR endpoints in similar fashion. Nasal lavage fluid (NLF) and nasal biopsy material were used to measure host defense factors and virus quantity, as described below.

Subjects were 18–40 years old, healthy, and had a history of smoking defined as>0.5 pack/day. Subjects were instructed to avoid cruciferous vegetables (which contain SFN) and anti-inflammatory medications, including corticosteroids and non-steroidal anti-inflammatory drugs, during the study period. A history of either receiving influenza vaccine or having a documented influenza infection in the previous year was a criterion for exclusion. Additionally, a cohort of healthy nonsmokers with otherwise identical inclusion and exclusion criteria were studied.

Both the pilot and the main study were approved by the UNC Biomedical Institutional Review Board. The main study was registered with ClinicalTrials.gov (Identifier: NCT01269723). All subjects gave written informed consent. The start date of the study was 1/24/2011 and the end date was 4/8/2013.

### Preparation of sprout homogenates

BSH shakes were prepared similarly to a previously published method [16]. One daily “dose” was 200 grams of BSH,containing approximately 111 grams fresh sprouts (about one 4-ounce package of Broccosprouts (Brassica Protection Products LLC)). The homogenates wereprepared by our clinical/translational research center'sNutrition Research and Metabolism Core, and were homogenized with water using a ratio of 1∶1.2 in a clean blender. The same weight of alfalfa sprouts, which contain minimal SFN, was prepared in an identical manner for the placebo (ASH) treatment. Both BSH and ASH were prepared from single lots of sprouts to insure uniform SFN dosing for the study, and stored at −20°C in aliquots, and thawed for ingestion the day prior to use. BSH and ASH shakes were labelled with subject number only, to maintain blinding of subjects and research staff administering the shakes.

### Nasal lavage and biopsy

Nasal lavagewas performed according to a method we have previously described [10] by repetitive spraying of sterile normal saline irrigation solution (4 ml total) into the nostril, followed by voluntary expelling of fluid by the subject into a specimen collection cup. Both nostrils werelavaged in this way and the resulting nasal lavage fluid (NLF) from both sides was combined. NLF was centrifuged at 500*g*×7 minutes to remove cells, and the cell-free supernatant stored in aliquots at −80°C until used in mediator assays. Total RNA was isolated from the cell pellets and analyzed for the expression of antioxidant, inflammatory, and immune genes as described below.

To biopsy nasal epithelium, cells were gently scraped from the inferior surface of the middle turbinate on each side of the nose, using a sterile plastic curette (Rhino-Probe, Arlington Scientific, Arlington, TX). After determination of cell differential (by morphology on Wright stain) and viability, specimens having >85% viable epithelial cells were used for immediate processing for qRT-PCR.

### Urine cotinine

Urine cotinine was measured by ELISA (Bio-Quant, Inc., San Diego, CA) and expressed as a ratio to creatinine, measured by a colorimetric assay (Oxford Biomedical Research, Rochester Hills, MI).

### Cytokine assays

Concentration of IL-6, IP-10, and IL-8 in the cell-free nasal lavage fluid was determined by enzyme-linked immunosorbent assay (ELISA) according to the manufacturer instructions (BD Biosciences, San Jose, CA).

### Influenza sequence quantitation and HMOX1 and NQO1 expression in NLF cells and nasal biopsy tissue by quantitative RT-PCR

RNA isolation from NLF cells and nasal biopsy tissue stored in Trizol, first-strand cDNA synthesis, and qRT-PCR were performed as previously described [20]. For RT-PCR analysis, primers for 18S, β-actin, (endogenous loading control) and HMOX1, and NQO1 were commercially available (Applied Biosystems, Grand Island, NY). Primers were designed for Influenza A and Influenza B Matrix 2 genes as follows: Influenza A Forward 5′-GGGTGCAGATGCAACGAT-3′, Reverse 5′-AATATCAAGTGCAAGATCCCAATG-3′, Probe 5′-I56-FAM/TGACCCTCTTGTTGTTGCCGC-3′; Influenza B Forward 5′-GCAAGTAAAACTAGGAACGCTCTGT-3′, Reverse 5′-CTGAAGATCTTGCTGCTCTGCTAT-3′, Probe 5′-I56-FAM/CGAGAAACAAGCATCACATTC-3′ (Integrated DNA technologies, Coralville, IA). qRT-PCR was performed using and Applied Biosystems 7500 Real Time PCR system. Gene expression was determined by the ΔΔCt method using 18S expression as an endogenous control for NLF cell samples and β-actin as an endogenous control for nasal biopsy tissue samples.

### Statistical analysis

The target sample size for the study was derived by estimating the number of subjects needed to detect a significant effect of BSH on NLF IL-6 response to LAIV, using area under curve data from our previous study in smokers[10]. Based on this, the minimum sample size required to detect a treatment effect of 150% of placebo was estimated to be 17 subjects per treatment group.

The study's primary endpoint was IL-6 in NLF after LAIV inoculation, expressed as area under curve (AUC) for ratio of IL-6 (Days 1–3) to baseline (Day 0), in smokers. AUC was compared between BSH and ASH groups using two-sample Student's t test [21]. Raw data were log-transformed in order to achieve normality required for t test. Secondary endpoints were analyzed in similar fashion and included influenza B RNA sequence quantity in NLF cells; HO-1 and NQO1 mRNA in NLF cells; tolerance of BSH; NLF cytokines other than IL-6 (IL-8, IP-10, IFN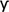); and nasal biopsy influenza B RNA sequence quantity and antioxidant enzyme mRNA. P<0.05 was considered statistically significant throughout.

The study was also carried out in a healthy nonsmoking young adult cohort for descriptive purposes. Data for nonsmokers were analyzed similarly as for smokers, but nonsmokers were studied with a different vaccine composition and were therefore not formally compared to smokers in the data analysis. All endpoints in the nonsmoker cohortwere treated as secondary analyses, separately from smokers.

## Results

### Pilot study

To confirm that our broccoli sprout homogenate (BSH) preparation would upregulate antioxidant enzymes in nasal cells similarly to the previously published study of Riedl et al. [16], we initially conducted a pilot study in which 10 healthy young adult volunteers underwent nasal lavage then ingested 1 BSH “shake” (see preparation method below) per day for 3 consecutive days. Expression of HMOX1 in NLF cells was measured by qRT-PCR. There was significantly increased median HMOX1 expression by the third day (median 3.5 fold increase over baseline; interquartiles 2.5, 9.0) (P = .02). The magnitude of increase in expression appeared to be related to baseline levels, such that subjects with lower baselines had greater increases (Spearman R = -0.73, P = 0.03). The majority of subjects reported mild gastrointestinal symptoms including abdominal fullness, bloating or flatulence. No severe symptoms were reported by any subject.

### Subject characteristics

**Figure 1:**
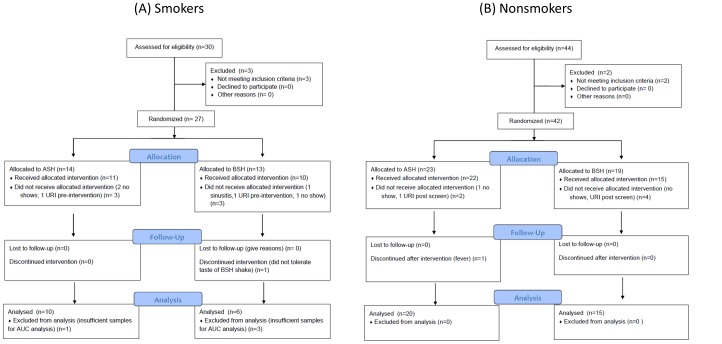
Flow diagram for recruitment and randomization of subjects for the (A) smokers and (B) nonsmokers cohorts in this study. URI  =  upper respiratory illness.

Study flow diagrams for the phases of recruitment, allocation, and data analysis for the smokers and nonsmokers cohorts are shown in Fig. 1. Smokers were more difficult to recruit and retain in the protocol than were nonsmokers. Among smokers who received the ASH or BSH intervention, 16/21 (76%)completed sufficient attendance and samples for final area under curve data analysis. By comparison, in nonsmokers35/37 (95%)of subjects who received ASH or BSH intervention had sufficient data for final analysis.

For the main randomized study, the demographic characteristics of subjects for final data analysis are shown in Table 1. Within each of the two separate study cohorts (smokers and nonsmokers), there was symmetric distribution for age, gender, race and BMI between treatment groups. Urine cotinine/creatinine ratios among the smokers at the screen visit were not statistically different between the ASH and BSH groups (P = 0.69), and were at levels consistent with a history of active smoking [10](Table 1).

**Table 1:** Demographic characteristics of subjects completing the randomized controlled study. Data are in mean ± SE except where indicated otherwise.

Cohort	Treatment arm	Age (yr)	Gender^1^	BMI	Race^2^	Urine cotinine^3^
Smoker	ASH(N = 10)	28.1±1.3	3/10	27.6±2.0	4/5/1	194±31
	BSH(N = 6)	27.3±1.7	2/6	27.2±1.4	4/2/0	313±132
Nonsmoker	ASH(N = 20)	26.9±1.3	14/20	24.9±0.8	15/5/0	N/A
	BSH(N = 15)	26.0±1.3	7/15	25.5±0.9	10/4/1	N/A

1 Female/total

2 White/African American/Asian

3 expressed as ng cotinine x 100/mg creatinine at Screen visit

Smokers were studied during the 2010-11 and 2011-12 vaccine “seasons” and thus received LAIV containing the influenza strains A/California/7/09 (H1N1)-like virus (pandemic (H1N1) 2009 influenza virus), A/Perth/16/2009 (H3N2)-like virus, and B/Brisbane/60/2008-like virus. One additional smoker completed the protocol in 2012-13, and thus received a vaccine with different H3N2 (A/Victoria/361/2011-like) and B (B/Wisconsin/1/2010-like) strains.Completion of the study by smokers fell short of the study target goal, due to a combination of relatively low smoking rates among local healthy young adults, poor compliance with the BSH supplementation protocol, and a high dropout rate after enrollment. Of 27 smokers who consented to participate, 11 (41%) did not complete the study for reasons including the development of illness before Day 0 or failure to return for all study visits. One subject did not tolerate the taste of the broccoli shakes and withdrew. Thus, 16 smokers completed the study.

Nonsmokers were studied in the 2012-13 vaccine season and thus received LAIV containing the following influenza strains: A/California/7/2009-like (pH1N1), A/Victoria/361/2011-like (H3N2), and B/Wisconsin/1/2010-like. Of 42 nonsmokers who consented to participate, 7 (17%) withdrew or were disqualified. None reported intolerable taste or side effects from the shakes. Thus, 35 nonsmokers completed the study.

### Effect of BSH on cytokines in NLF

**Figure 2:**
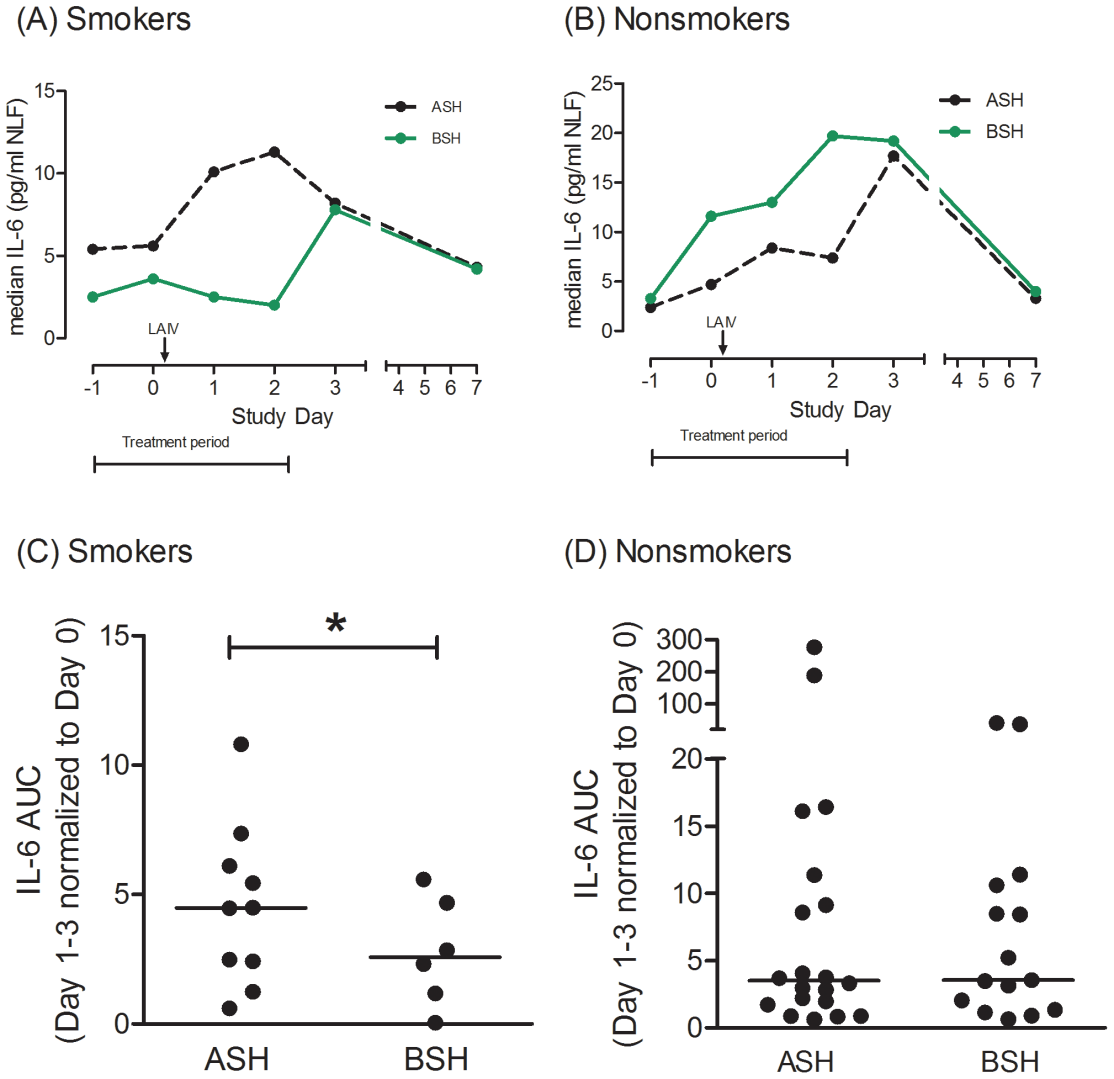
IL-6 in nasal lavage fluids (NLF) and IL-6 area under curve (AUC) for Days 1–3 normalized to Day 0, for smoker and nonsmoker study groups. Data for raw IL-6 in NLF (A, B) are shown as medians on each study day, for each treatment group. Data for AUC (C, D)are for individual subjects; bar indicates median for treatment group. During the “Treatment period” subjects received broccoli sprout homogenates (BSH) or alfalfa homogenates as control (ASH) once daily. Live attenuated influenza virus (LAIV) was inoculated nasally on Day 0, after nasal lavage. * P<.05 for BSH vs. ASH.

We analyzed the effect of BSH vs. ASH on nasal cytokines prior to LAIV (Day −1 vs. Day 0), and after LAIV (area under curve for Days 1–3 normalized to Day 0). Table 2 shows summarized NLF cytokine data for smokers. The BSH group did not show a statistically significant effect on pre-LAIV (Day −1 vs. day 0) levels of any the cytokines assayed, compared to ASH. After LAIV, in both treatment groups, IL-6 and IP-10 tended to increase over Days 1–3, then declined to baseline levels by Day 7; and there was little change in IL-8. These trends in response to LAIV were consistent with our prior study in smokers [10]. Levels of IFN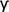 were near the low end of the quantifiable range throughout the study period (data not shown). BSH had no statistically significant effect on the IL-8, IP-10, or IFN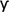 responses to LAIV. However, in smokers, BSH was associated with significantly reduced IL-6 response to LAIV (_log_AUC 2.13±0.79), compared to placebo (_log_AUC3.27±0.99) (P = 0.03) (Fig. 2). Fig. 2A suggests that this reduction was mainly due to lack of an IL-6 response on study days 1–2.

**Table 2:** Nasal lavage fluid (NLF) cytokines in Smokersand Nonsmokers after LAIV

Cohort	Cytokine	Arm	Day 0	Day 1	Day 2	Day 3	Day 7
Smokers	IL-6	ASH	5.6 (3.1, 16.0)	10.1(5.1, 28.0)	11.3(7.8, 20.5)	8.2(5.0, 38.5)	4.3(2.9, 19.3)
		BSH	3.6(2.1, 29.2)	2.5(1.5, 3.9)	2.0(1.2, 12.3)	7.8(3.1, 13.2)	4.2(2.4, 9.8)
	IP-10	ASH	1572(945, 5687)	3366(1789, 6323)	5424(2797, 10787)	6454(2370, 12348)	2119(1761, 3238)
		BSH	2531(1517, 3914)	2021(867, 6512)	6101(2864, 17838)	6008(2924, 10586)	2720 (2025, 4307)
	IL-8	ASH	2436(1623, 3229)	2910(2152, 4156)	2559(1753, 3682)	2690(1870, 5139)	2305(1840, 3041)
		BSH	4428(2068, 7537)	2707(1783, 3813)	3788(2582, 5693)	2359(2047, 5010)	3855(2468, 6159)
Nonsmokers	IL-6	ASH	4.5(2.1, 24.5)	11.4(1.4, 27.2)	10.3(1.9. 33.2)	19.3(4.4, 47.4)	3.3(1.2, 14.7)
		BSH	10.6(3.0, 36.6)	13.0(2.1, 47.7)	19.7(7.6, 54.2)	19.2(5.0, 69.4)	4.0(2.5, 46.5)
	IP-10	ASH	3039(2367, 6555)	2950(2198, 11490)	6945(3689, 24305)	9406(3935, 22272)	4504(2669, 20810)
		BSH	2954(1362, 7647)	3053(1659, 4061)	5445(2028, 15506)	4980(2277, 16170)	6416(2211, 9813)
	IL-8	ASH	1550(562, 3232)	2891(974, 6345)	2094(982, 5323)	3235(1145, 6815)	2162(795, 3726)
		BSH	2717(2152, 4776)	3735(2093, 5599)	2291(1691, 4851)	2326(1951, 5244)	2507(1701, 4880)

.Subjects received BSH or placebo on Days −1, 0, 1, and 2; LAIV was administered on Day 0 after nasal lavage. Data (pg/mL NLF) are shown as median (interquartiles). Treatment arms: ASH  =  alfalfa sprout homogenate (control), BSH  =  broccoli sprout homogenate.

Table 2 also shows summarized NLF cytokine data for nonsmokers. At baseline (prior to LAIV), BSH was associated with a statistically significant but small decrease in IL-8 in nonsmokers. Otherwise, BSH did not have a statistically different effect on baseline (pre-LAIV) levels of the factors assayed, between Day (−1) and Day 0, compared to ASH controls. In both treatment groups, consistent with our prior study[10], IL-6 and IP-10 both increased over Days 1–3 following LAIV inoculation, then declined to baseline levels by Day 7, and there was little change in IL-8. IFN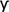 was again low for most subjects at most time points (data not shown). The BSH effect on IL-6 noted in smokers did not occur in nonsmokers, for whomthere were no statistically significant effects of BSH compared to control on any of the post-LAIV cytokines assayed, in the AUC analyses (Fig. 2).IL-6 levels and responses to LAIV were generally lower in smokers than in nonsmokers, also consistent with our prior observations [10].

### Effect of BSH on influenza quantity in NLF cells and nasal biopsies

**Figure 3:**
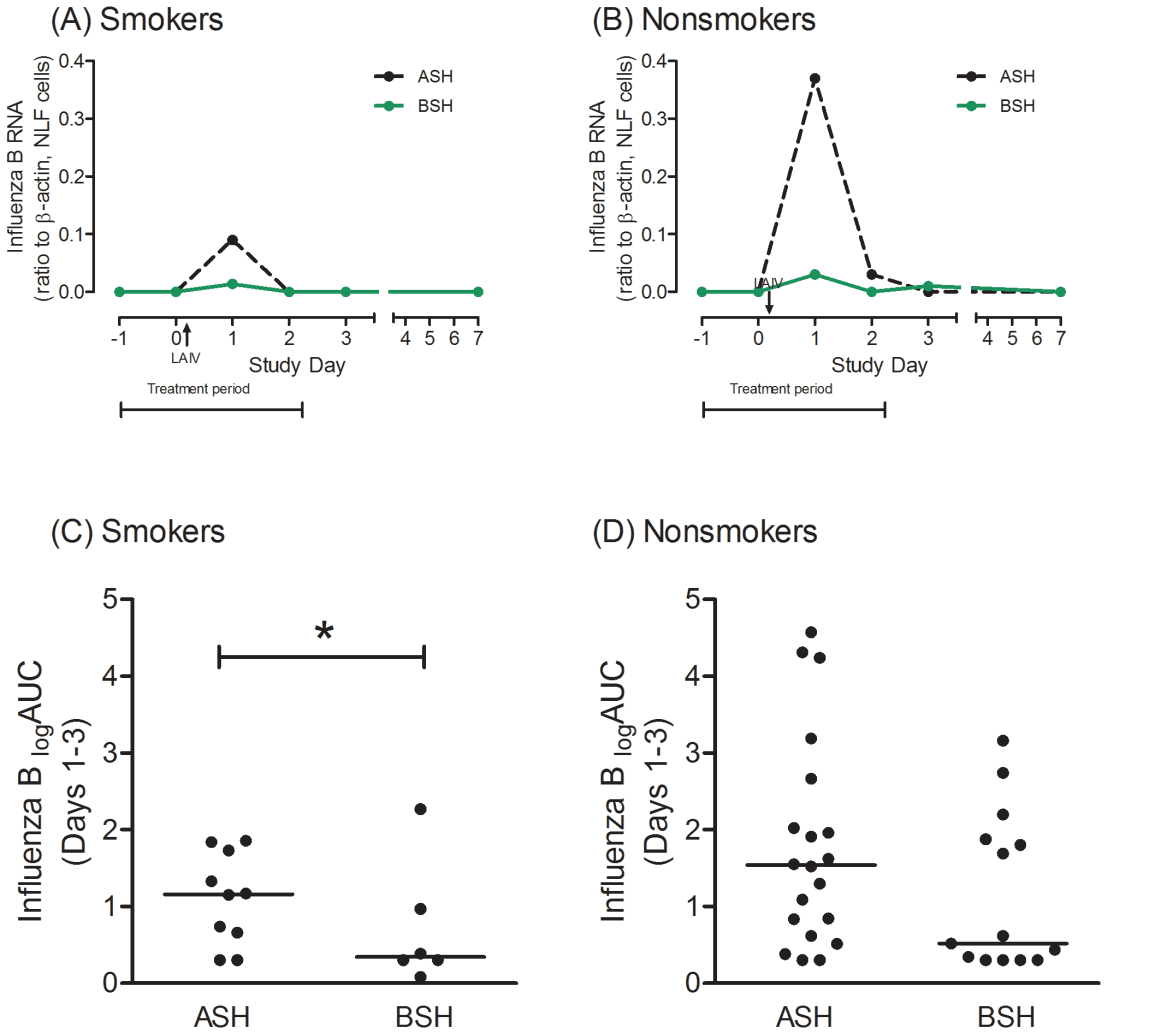
Influenza B RNA sequence quantity in nasal lavage fluid cells for (A) smokers and (B) nonsmokers. Data are shown as medians on each study day, for each treatment group. During the “Treatment period” subjects received broccoli sprout homogenates (BSH) or placebo control (alfalfa sprout shakes) once daily. Live attenuated influenza virus (LAIV) was inoculated nasally on Day 0, after nasal lavage. (C) and (D) are area under curve data (Day 1–3, normalized to Day 0 baseline) for smokers and nonsmokers respectively. Bar indicates median for treatment group. * P<.05 for BSH vs. ASH (AUC).

In NLF cells in smokers, no virus sequences were detectable in any subject in either treatment group, at the Day (−1) or Day 0 baselines, as expected; and other than on Day 1, the majority of samples had undetectable levels of viral RNA. However, as shown in Fig. 3, the BSH group had significantly reduced influenza B sequence quantity (_log_AUC −3.67±0.77), compared to the placebo group (_log_AUC −1.94±1.40)(P = 0.01). In nasal epithelial biopsies obtained at study screening there were no viral sequences detected in either treatment group, as expected. At Day 2, no viral sequences were detected in any subject in the BSH group, whereas influenza B sequences were detected in 2 of 10 controls, but the effect was not statistically significant (data not shown).

In nonsmokers, a similar pattern of reduced virus sequences in NLF cells was observed in the BSH group (Fig. 3).While the median reduction in virus quantity appeared to be even greater than that seen in the smokers (_log_AUC −1.88±2.36 for BSH compared to −0.49±1.33 in controls), the variability was greater and the effect was not statistically significant (P = 0.15).

### Effects of BSH on expression of HMOX1 and NQO1 in NLF cells and nasal biopsies

Data for expression of antioxidants HMOX1 and NQO1 in NLF cells on each study day are shown in Table 3 for smokers. BSH had no statistically significant effect on baseline (pre-LAIV) expression of HMOX1 or NQO1, and the HMOX1 response to LAIV as measured by AUC was not significantly altered by BSH. NQO1 response to LAIV in NLF cells, as measured by AUC for Days 1–3 normalized to Day 0, was significantly increased in the BSH group (54.4±47.4) compared to ASH (23.2±20.2) (P = 0.04). Inspection of the raw data medians for time course of NQO1 in NLF cells in smokers (Table 3) suggests that this was because the Day 0 baseline was lower in the BSH group, but the Day (−1) level prior to BSH treatment also appeared to be lower in this group. A similar pattern in the raw data was noted for nonsmokers (Table 3), but there was no significant difference between treatment groups in the AUC analysis.

**Table 3:** Nasal lavage fluid (NLF) cell expression of antioxidant enzymes in Smokersand Non-smokers after LAIV

Cohort	Endpoint	Arm	Day 0	Day 1	Day 2	Day 3	Day 7
Smokers	HMOX1	ASH	1.97(0.98, 6.60)	2.33(1.23, 5.32)	1.78(1.21, 5.42)	1.97(0.72, 5.27)	2.11(0.91, 4.31)
		BSH	0.39(0.29, 3.10)	1.29(0.93, 2.34)	1.58(1.01, 3.95)	0.90(0.63, 3.33)	0.86(0.32, 2.20)
	NQO1	ASH	1.70(0.82, 5.48)	2.33(1.23, 5.32)	1.76(0.67, 4.00)	1.47(0.63, 5.15)	2.11(0.91, 4.31)
		BSH	0.30(0.14, 1.95)	1.29(0.93, 2.34)	1.58(1.01, 3.95)	0.90(0.63, 3.33)	0.86(0.32, 2.20)
Nonsmokers	HMOX1	ASH	2.21(1.00, 5.62)	1.57(0.58, 3.71)	1.18(0.77, 4.34)	0.98(0.50, 1.74)	1.75(0.89, 3.43)
		BSH	0.68(0.42, 2.05)	0.91(0.46, 1.87)	1.03(0.64, 2.03)	0.59(0.42, 1.08)	0.77(0.33, 1.52)
	NQO1	ASH	2.211.00, 5.62)	1.32(0.56, 3.60)	1.18(0.77, 4.34)	0.98(0.50, 1.74)	1.75(0.89, 3.43)
		BSH	0.68(0.42, 2.05)	0.91(0.46, 1.87)	0.96(0.45, 1.99)	0.56(0.38, 1.00)	0.77(0.33, 1.52)

.Subjects received BSH or placebo on Days −1, 0, 1, and 2; LAIV was administered on Day 0 after nasal lavage. Data are shown as median (interquartiles). Treatment arms: ASH  =  alfalfa sprout homogenate (control), BSH  =  broccoli sprout homogenate.

To assess the effect of BSH on epithelial expression of HMOX1 and NQO1 in the setting of LAIV infection, the ratio of Day 2 to screen day mRNA expression was measured in the nasal biopsies and compared between the treatment groups. In smokers, the median HMOX1 expression ratio was higher in the BSH group (Table 4) but this was not statistically significant (P = 0.36). There was no statistically significant BSH effect con NQO1 compared to control in smokers. There was no statistically significant effect of BSH on epithelial expression of HMOX1 or NQO1 for nonsmokers. Epithelial expression of NQO1 tended to be higher in the smokers than in the nonsmokers(Table 4), both at baseline and after LAIV.

**Table 4:** Effect of BSH and control treatments on HMOX1 and NQO1

Cohort	Endpoint	Arm	Screen	Day 2	Treatment effect (Day 2/Screen)
Smokers	HMOX1	ASH	1.18 (0.84, 3.41)	0.90 (0.60, 1.53)	0.92(0.21, 2.21)
		BSH	1.09 (0.76, 2.86)	2.09 (0.91, 2.93)	1.49(0.80, 2.24)
	NQO1	ASH	0.35 (0.23, 0.47)	0.36 (0.21, 0.46)	0.99(0.62, 1.42)
		BSH	0.42 (0.26, 0.66)	0.53 (0.28, 0.72)	0.93(0.76, 1.88)
Nonsmokers	HMOX1	ASH	0.93 (0.56, 1.38)	0.88 (0.53, 1.73)	1.01(0.56. 1.84)
		BSH	0.76 (0.39, 1.90)	1.23 (0.59, 1.79)	1.14(0.65, 2.34)
	NQO1	ASH	0.14 (0.10, 0.22)	0.15 (0.11, 0.26)	1.13(0.85, 1.89)
		BSH	0.13 (0.09, 0.20)	0.17 (0.13, 0.27)	1.37(0.93, 1.63)

Data are shown as median (interquartiles). Treatment arms: ASH  =  control (alfalfa sprout homogenate), BSH  =  broccoli sprout homogenate.

**Figure 4:**
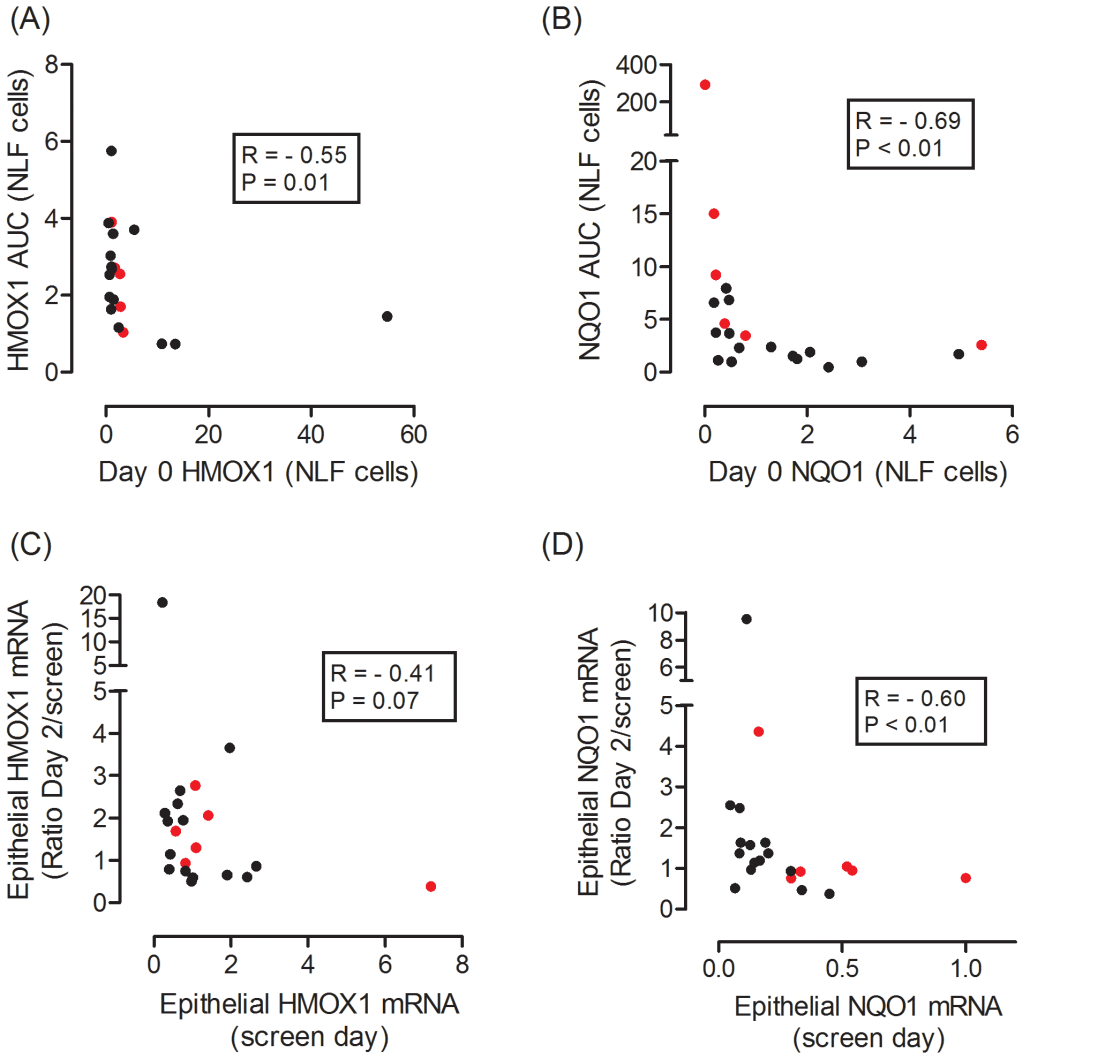
Relationship of baseline (screen day) mRNA quantity of antioxidant enzymes cells to magnitude of change in mRNA quantity after LAIV, in subjects who received BSH. Data are shown for (A) HMOX1 and (B) NQO1 in NLF cells (AUC for Days 1–3 normalized to baseline), and for (C) HMOX1 and (D) NQO1 in nasal epithelial biopsy cells (ratio of Day 2 biopsy to screen day). Data for smokers are shown as red dots and nonsmokers as black dots. R and P values are for Spearman correlation coefficient, for smokers and nonsmokers who received BSH, considered together.

Given the apparent relationship between baseline HMOX1 expression in NLF cells and subsequent response to BSH in the pilot study, for descriptive purposes we assessed the correlation between baseline expression and subsequent response to BSH, for HMOX1 and NQO1 (Fig. 4). For both NLF cells (Fig. 4 A, B) and nasal biopsy cells (Fig. 4 C,D), lower initial (screen day) expression of the antioxidant enzymeswere associated with greater “responses” to BSH.

## Discussion

Smokers are at increased risk for influenza infection [3]–[8], [22] and we have previously shown that their nasal mucosal IL-6 and IP-10 responses to LAIV are blunted [10]. In a randomized study of young adult smokers, we have shown here that short term ingestion of a nutritional supplement intended to upregulate antioxidant levelssignificantly reduces IL-6 response and virus quantity, while possibly enhancing NQO1 expression in epithelium. Based on our prior *in vitro* studies in nasal epithelial cells [11], [13], we hypothesized that BSH reduced viral replication by reducing oxidative stress in nasal cells, resulting in secondary reduction in downstream host defense responses including IL-6 production. Some of these results, particularly suppression of virus quantity, appeared to show a similar tendency in a cohort of healthy nonsmokers studied separately, though the results were not statistically significant.

Airway tissues from smokers are under chronic oxidative stress [23]and a relationship between cellular oxidative stress and susceptibility to respiratory virus infection has been described previously. Macchia et al. reported that inhibition of glutathione (GSH) synthesis in epithelial cells leads to increased replication of Sendai virus. [24] In our own studies comparing differentiated nasal epithelial cells from smokers and nonsmokers, we have reported both increased influenza replication and suppression of antiviral pathways in cells from smokers.[11] We have also found that treatment of human respiratory epithelium with *Nrf2*-activating agents including SFN and EGCG, as well as GSH,inhibits influenza replication in nasal epithelial cells; the mechanisms *in vitro* appear to include both upregulation of antiviral pathway elements and increased ratio of antiproteases to proteases, which in turn inhibits the proteolytic activation of influenzahemagglutinin, which is necessary for initial binding and entry of the virus. [13], [25] Animal models have also suggested that manipulation of *Nrf2* activation can affect viral replication. Cho et al reported that *Nrf2^−/−^* mice had increased pulmonary inflammation and severity of disease compared to wild type, and pretreatment with SFN reduced illness in *Nrf2^+/+^* mice[26]. Finally, in a murine model of tobacco smoke-induced oxidative stress, the “mucoregulatory” compound S-carboxymethylcysteine reduced both influenza replication and pulmonary inflammation, while activating *Nrf2.* [27]Thus, Nrf2-enhancing supplementation strategies may reduce susceptibility to respiratory virus infections, especially in populations under chronic oxidative stress, such as smokers.

Broccoli sprouts have been previously investigated as a concentrated source of precursors of the potent *Nrf2* inducingisothiocyanate compound sulforaphane (SFN)[28]. In humans, SFN has been studied as a chemopreventive agent[29], as a phase II enzyme inducer to protect against the pro-inflammatory effects of diesel exhaust particles in airway epithelium [30], and is currently being examined in the context of COPD (NCT01335971), based on the findings that upregulating*Nrf2* may inhibit progression of the disease [31], [32]. Riedl et al.[16] reported that ingestion of broccoli sprout homogenates resulted in dose-dependent increased expression of Phase II enzymes over baseline, including HMOX1 and NQO1, in NLF cells from human volunteers, an effect not seen with alfalfa sprouts; the BSH and control preparations and “doses” we used in the present study were modeled after this. It was also recently reported by Heber et al.[18] that broccoli sprout extracts at a similar dose, given for 4 days, had a suppressive effect on diesel particle-induced allergic inflammation in volunteers with allergic rhinitis. Hence, SFN-containing broccoli sprout extracts have been examined in relation tochronic inflammatory lung diseases and additional clinical trials are underway assessing the potential of this “nutraceutical” in this context.

In addition to our data shown here, several randomized, placebo-controlled studies of the effect of nutritional or antioxidant supplements on influenza infection or vaccine effectiveness have been reported. For example, Vitamin D3 supplementation was associated with a reduction in antigen-positive influenza A infection from 18% to 10% in school children[33], and may have decreased the incidence of asthma exacerbation. In a double-blind randomized trial, cranberry juice, which has high concentration of polyphenols and antioxidant activity, was consumed daily for 10 weeks and resulted in reduced cold and flu symptoms as well as markedly enhanced proliferation of peripheral blood 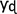-T cells after PHA stimulation *ex vivo* [34]. In another double-blind placebo-controlled study, fucoidan, a sulfated polysaccharide extracted from seaweed, was shown to enhance antibody production to trivalent influenza vaccine in elderly Japanese volunteers, possibly associated with enhanced NK cell activity[35]. An herbal mixture of Echinacea, propolis, and Vitamin C taken for 12 weeks reduced upper respiratory infections in children[36]. Green tea catechins and threonine taken in capsule form reduced the incidence of clinically-defined influenza illnesses in Japanese health care workers from 13% to 4% [37]. Our study thus adds to a small but growing body of randomized controlled studies supporting the concept that nutritional factors can alter specific host defense responses and could thus potentially play an important role in reducing the severity of influenza.

We sought evidence for upregulation of the *Nrf2*-dependent antioxidant enzymes HMOX1 and NQO1 in nasal cells in our BSH treated volunteers. While there was a statistically significant elevation in AUC for NQO1 in NLF cells of smokers treated with BSH, we did not see a significant effect of BSH on HMOX1. It is possible our subject population was heterogeneous for baseline oxidant stress. Though our study was not designed to test dose response or pre-existing “deficiency” of antioxidants, the impact of BSH on expression of HMOX1 and NQO1 after infection may have been linked to baseline levels, i.e. the lower the starting levels of expression, the greater the impact. Interestingly, this pattern appeared to be present in both smokers and nonsmokers (Fig. 4). It seems plausible that nutritional interventions aimed at boosting antioxidants may be most effective in individuals who are relatively antioxidant-deficient at baseline, a condition likely to be more prevalent in smokers. [23]

Our study had several limitations. The infection induced by the LAIV vaccine is inherently limited as a model for natural infection, since the vaccine is specifically designed to have only limited replication in the upper airway, a feature which makes the model safe but places constraints on how generalizable or clinically relevant the study conclusions are for community-acquired influenza infections. Since our purpose was to study LAIV as a model infection rather than as a vaccine, we focused on shorter-term innate immunity endpoints, and did not assess longer term effects of BSH on immune responses to LAIV. To minimize altered responses to LAIV due to prior influenza exposures, we excluded subjects with a history of either vaccination or influenza in the past 12 months, but serologic screening could have confirmed an immunologically naïve subject population. We had relative difficulty with recruitment and retention of subjects in the smokers cohort, resulting in a smaller N than originally targeted.

Ingestion of the kind of BSH used in the study described here may not be feasible to enhance mucosal host defense in the general public; however, based on our *in vitro* studies[11], [13] activation of *Nrf2*-dependent pathways are mechanistically linked to the SFN-induced protection against respiratory virus infection, suggesting that other *Nrf2*-activating agents may have similar effects. For example, the benefit of synthetic or naturally occurring triterpenoids, which have significantly greater potency in activating *Nrf2* [38], may need to be explored in the context of viral infection.

Despite some of thelimitations described above, we believe our study to be the first randomized, placebo controlled trial suggesting a measurable effect of a short-term nutritional intervention on specific mucosal host defense responses to influenza, and perhaps virus replication, in humans.Our study focused on specific host defense factors using a relatively artificial model, and its clinical significance is unclear. However, our results are encouraging as proof of concept for further study of the strategy of developing nutritional antioxidants as an additional, low-costand low-risk measure for reducing the impact of influenza. This may be especially true for subgroups with underlying risk factors related to chronic smoke exposure, pre-existing poor nutrition, or underlying disease. While our study was not designed to directly compare the effects of broccoli sprouts in smokers vs. nonsmokers, we speculate that nonsmokers with relative antioxidant deficiency could also benefit from this approach.

## Supporting Information

Checklist S1 **This is the CONSORT 2010 checklist of information included in the manuscript for a randomized trial.**
(DOC)

Protocol S1 **This is a PDF file of the approved IRB protocol for this study.**
(PDF)
