# Correction: Effect of Broccoli Sprouts on Nasal Response to Live Attenuated Influenza Virus in Smokers: A Randomized, Double-Blind Study

**DOI:** 10.1371/journal.pone.0109513

**Published:** 2014-09-26

**Authors:** The PLOS ONE Staff

Table 3 incorrectly contains duplicated data in multiple cells of the HMOX1 rows. The authors have provided a corrected version of Table 3 below.

**Table 3:** Nasal lavage fluid (NLF) cell expression of antioxidant enzymes in Smokers and Non-smokers after LAIV.

Cohort	Endpoint	Arm	Day 0	Day 1	Day 2	Day 3	Day 7
**Smokers**	HMOX1	ASH	2.44 (1.07, 4.90)	2.43 (1.07, 6.24)	2.11 (0.71, 6.86)	2.74 (0.97, 5.14)	1.73 (0.84, 3.82)
		BSH	2.73 (1.44, 3.13)	2.14 (1.49, 2.37)	2.83 (1.98, 3.93)	1.49 (1.27, 2.97)	2.05 (1.12, 3.27)
	NQO1	ASH	1.70 (0.82, 5.48)	2.33 (1.23, 5.32)	1.76 (0.67, 4.00)	1.47 (0.63, 5.15)	2.11 (0.91, 4.31)
		BSH	0.30 (0.14, 1.95)	1.29 (0.93, 2.34)	1.58 (1.01, 3.95)	0.90 (0.63, 3.33)	0.86 (0.32, 2.20)
**Nonsmokers**	HMOX1	ASH	1.48 (0.76, 2.79)	1.07 (0.65, 4.68)	1.33 (0.78, 2.74)	1.43 (1.06, 3.30)	1.24 (1.04, 2.60)
		BSH	1.15 (0.90, 5.51)	2.02 (1.06, 3.47)	1.58 (0.97, 4.56)	1.70 (1.21, 3.46)	1.19 (0.98, 2.49)
	NQO1	ASH	2.21 1.00, 5.62)	1.32 (0.56, 3.60)	1.18 (0.77, 4.34)	0.98 (0.50, 1.74)	1.75 (0.89, 3.43)
		BSH	0.68 (0.42, 2.05)	0.91 (0.46, 1.87)	0.96 (0.45, 1.99)	0.56 (0.38, 1.00)	0.77 (0.33, 1.52)
