# Supplementary material for: Effect of Broccoli Sprouts on Nasal Response to Live Attenuated Influenza Virus in Smokers: A Randomized, Double-Blind Study
Source: PLoS One. 2014 Jun 9;9(6):e98671. doi: 10.1371/journal.pone.0098671 (PMC4049587; doi:10.1371/journal.pone.0098671)
Supplement: Protocol S1 — This is a PDF file of the approved IRB protocol for this study. (PDF) [file pone.0098671.s002.pdf]

OFFICE OF HUMAN RESEARCH ETHICS
Institutional Review Board

APPLICATION FOR IRB APPROVAL OF
HUMAN SUBJECTS RESEARCH
Version June 25, 2009

Part A.1. Contact Information, Agreements, and Signatures

Date: 10-1-10

Title of Study: Effects of sulforaphane on innate immune responses to live attenuated influenza virus in smokers and nonsmokers

Name and degrees of Principal Investigator: Terry L. Noah, MD
Department: Pediatrics and CEMALB Mailing address/CB #: 7310
UNC-CH PID: 7042-78684 Pager: 216-2961
Phone #: 966-1055 Fax #: 966-6179 Email Address: terry_noah@med.unc.edu

For trainee-led projects: __ undergraduate __ graduate __ postdoc __ resident __ other
Name of faculty advisor:
Department: Mailing address/CB #:
Phone #: Fax #: Email Address:

Center, institute, or department in which research is based if other than department(s) listed above:

Name of Project Manager or Study Coordinator (if any): Peg Herbst, RN, MSN
Department: Center for Environmental Medicine, Asthma and Lung Biology
Mailing address/CB #: 7310 Phone #: 966-2879 Fax #: 6-9863
Email Address: Margaret_herbst@med.unc.edu

List **all other project personnel** including co-investigators, and anyone else who has contact with subjects or identifiable data from subjects. **Include name, location (UNC or specific outside location), role and email address for each person who should receive electronic copies of IRB correspondence to PI.**
Co-investigators: Ilona Jaspers, PhD, Matthew Kesic, PhD, Johnny Carson PhD
Project personnel: Michelle Hernandez MD, Marianne Muhlebach, MD, David Peden MD, Claire Cherazi MD, Paula Murphy, Luisa Brighton, Wenli Zhang, Martha Almond RRT, Aline Kala RN, Carole Robinette MS, Lynne Newlin-Clapp, BA

Name of funding source or sponsor (*please do not abbreviate*): National Institutes of Health/NHLBI
__ not funded _X_ Federal __ State __ industry __ foundation __ UNC-CH
__ other (specify):

For external funding, RAMSeS proposal number (from Office of Sponsored Research): 09-1339

For industry sponsored research (if applicable): NA

Sponsor's master protocol version #: Version date:

Investigator Brochure version #: Version date:

Any other details you need documented on IRB approval:

Checklist of Items to Include with Your Submission

Include the following items with your submission, where applicable.

- Check the relevant items below and include one copy of all checked items 1-11 in the order listed.
- Also include two additional collated sets of copies (sorted in the order listed) for items 1-6.

Applications must "stand alone" and should provide all information requested, i.e., complete answers must be contained in the application. While you may reference other documents with supporting information, do not respond solely by stating "see attached."

Applications will be returned if these instructions are not followed.

Check	Item	Total No. of Copies
X	1. This application. One copy must have original PI signatures.	3
X	2. Consent and assent forms (include DHHS-approved sample, when one exists), fact or information sheets, phone and verbal consent scripts.	3
X	3. HIPAA authorization addendum to consent form.	3
X	4. All recruitment materials including final copies of printed advertisements, audio/video taped advertisements, scripts, flyers, letters, and emails.	3
X	5. Questionnaires, focus group guides, scripts used to guide phone or in-person interviews, etc.	3
□	6. Documentation of reviews from any other committees (e.g., Clinical and Translational Research Center (CTRC), Oncology Protocol Review Committee, or local review committees in Academic Affairs).	3
X	7. Protocol, grant application or proposal supporting this submission, if any (e.g., extramural grant application to NIH or foundation, industry protocol, student proposal). This must be submitted if an external funding source or sponsor is checked on the previous page.	1
□	8. Addendum for Multi-Site Studies where UNC-CH is the Lead Coordinating Center.	1
□	9. Data use agreements (may be required for use of existing data from third parties).	1
X	10. Only for those study personnel *not* in the online UNC-CH human research ethics training database (http://cfx3.research.unc.edu/training_comp/): Documentation of required training in human research ethics. *Please note that the ethics training documentation for Dr. Claire Cherazi (her current married surname) is included here, but it was completed under her maiden name Van Eenwyk.	1
□	11. For drug studies, Investigator Brochure if one exists. If none, include package insert for previously approved uses..	1

Principal Investigator: I will personally conduct or supervise this research study. I will ensure that this study is performed in compliance with all applicable laws, regulations and University policies regarding human subjects research. I will obtain IRB approval before making any changes or additions to the project. I will notify the IRB of any other changes in the information provided in this application. I will provide progress reports to the IRB at least annually, or as requested. I will report promptly to the IRB all unanticipated problems or serious adverse events involving risk to human subjects. I will follow the IRB approved consent process for all subjects. I will ensure that all collaborators, students and employees assisting in this research study are informed about these obligations. All information given in this form is accurate and complete.

____ _______________
Signature of Principal Investigator Date

Faculty Advisor if PI is a Student or Trainee Investigator: I accept ultimate responsibility for ensuring that this study complies with all the obligations listed above for the PI.

____ _______________
Signature of Faculty Advisor Date

Note: The following signature is not required for applications with a student PI.

Department or Division Chair, Center Director (or counterpart) of PI: (or Vice-Chair or Chair's designee if Chair is investigator or otherwise unable to review): I certify that this research is appropriate for this Principal Investigator, that the investigators are qualified to conduct the research, and that there are adequate resources (including financial, support and facilities) available. If my unit has a local review committee for pre-IRB review, this requirement has been satisfied. I support this application, and hereby submit it for further review.

____ _______________
Signature of Department Chair or designee Date

____ _______________
Print Name of Department Chair or designee Department

Part A.2. Summary Checklist *Are the following involved?*

	Yes	No
A.2.1. Existing data, research records, patient records, and/or human biological specimens?	__	_X_
A.2.2. Surveys, questionnaires, interviews, or focus groups with subjects?	_x_	_ _
A.2.3. Videotaping, audiotaping, filming of subjects, or analysis of existing tapes?	__	_X_
A.2.4. Do you have <u>specific plans</u> to enroll subjects from these vulnerable or select populations:		
a. UNC-CH students or UNC-CH employees?	__	_X_
b. Non-English-speaking?	__	_X_
c. Decisionally impaired?	__	_X_
d. Patients?	__	_X_
e. Prisoners, others involuntarily detained or incarcerated, or parolees?	__	_X_
f. Pregnant women?	__	_X_
g. Minors (less than 18 years)? ***If yes***, give age range: to years	__	_X_
A.2.5. a. Are sites outside <u>UNC-CH</u> <u>engaged</u> in the research?	__	_X_
b. Is UNC-CH the sponsor or <u>lead coordinating center</u> for a multi-site study?	__	_X_
If yes, include the ***<u>Addendum for Multi-site Studies</u>***.		
If yes, will any of these <u>sites be outside the United States</u>?	__	__
If yes, is there a local ethics review committee agency with jurisdiction? (provide contact information)	__	__
A.2.6. Will this study use a data and safety monitoring board or committee?	__	_X_
If yes: UNC-CH NC TraCS DSMB? (<u>must apply separately</u>)	__	__
Lineberger Cancer Center DSMC?	__	__
Other? Specify:	__	__
A.2.7. a. Are you collecting sensitive information such as sexual behavior, HIV status, recreational drug use, illegal behaviors, child/physical abuse, immigration status, etc?	_X_	__
b. Do you plan to obtain a federal Certificate of Confidentiality for this study?	_X_	__
c. Is this research classified (e.g., requires security clearance)?	__	_X_
A.2.8. a. <u>Investigational</u> drugs? (provide **IND #**)	__	_X_
b. Approved drugs for "non-FDA-approved" conditions?	__	_X_
All studies testing substances in humans must provide a letter of acknowledgement from the <u>UNC Health Care Investigational Drug Service</u> (IDS).		
A.2.9. Placebo(s)?	_X_	__
A.2.10. <u>Investigational</u> devices, instruments, machines, software? (provide **IDE #**)	__	_X_
A.2.11. Fetal tissue?	__	_X_
A.2.12. Genetic studies on subjects' specimens?	_X_	__
A.2.13. Storage of subjects' specimens for future research? ***If yes****, see instructions for* ***<u>Consent for Stored Samples</u>***.	_X_	__
A.2.14. Diagnostic or therapeutic ionizing radiation, or radioactive isotopes, which subjects would not receive otherwise? ***If yes****, approval by the* ***<u>UNC-CH Radiation Safety</u> Committee*** *is required.*	__	_X_
A.2.15. Recombinant DNA or gene transfer to human subjects? ***If yes****, approval by the* ***<u>UNC-CH Institutional Biosafety</u> Committee*** *is required.*	__	_X_
A.2.16. Does this study involve UNC-CH cancer patients? ***If yes****, submit this application directly to the* ***<u>Oncology Protocol Review Committee</u>***.	__	_X_
A.2.17. Will subjects be studied in the Clinical and Translational Research Center (CTRC) or is the CTRC involved in any other way with this study? If yes, obtain the ***<u>CTRC Addendum</u>*** *and submit completed application (IRB application and Addendum) directly to the CTRC. The CTRC includes facilities located on the 3rd floor of the Main Hospital (formerly GCRC) and Ground floor Burnett-Womack (formerly CCCT).*	_X_	__
A.2.18. Will gadolinium be administered as a contrast agent?	..__	.._X_
A.2.19. Will subjects' <u>Social Security Number</u> (SSN) be collected for:		

a. processing payments greater than $200 per year, to support IRS reporting (see also B.6)?<br>b. processing payments of any amount through UNC-CH Accounts Payable?<br>c. use as a unique identifier for study tracking purposes for national registry or database?	_X_<br>__<br>__	__<br>__<br>__

Part A.3. Conflict of Interest Questions and Certification

The following questions apply to **all investigators and study staff** engaged in the design, conduct, or reporting results of this project **and/or their immediate family members.** For these purposes, "family" includes the individual's spouse and dependent children. "Spouse" includes a person with whom one lives together in the same residence and with whom one shares responsibility for each other's welfare and shares financial obligations.

A.3.1. Currently or during the term of this research study, does any member of the research team or his/her family member have or expect to have:		
(a) A personal financial interest in or personal financial relationship (including gifts of cash or in-kind) with the sponsor of this study?	__ yes	_X_ no
(b) A personal financial interest in or personal financial relationship (including gifts of cash or in-kind) with an entity that owns or has the right to commercialize a product, process or technology studied in this project?	__ yes	_X_ no
(c) A personal financial interest in or personal financial relationship (including gifts of cash or in-kind) with an entity engaged in the performance of this project as a subcontractor, sub-recipient or vendor?	__ yes	_X_ no
(d) A board membership of any kind or an executive position (paid or unpaid) with the sponsor of this study or with an entity that owns or has the right to commercialize a product, process or technology studied in this project?	__ yes	_X_ no
A.3.2. Has the University or has a University-related foundation received a cash or in-kind gift from the sponsor of this study for the use or benefit of any member of the research team?	__ yes	_X_ no
A.3.3. Has the University or has a University-related foundation received a cash or in-kind gift for the use or benefit of any member of the research team from an entity that owns or has the right to commercialize a product, process or technology studied in this project?	__ yes	_X_ no

If the answer to ANY of the questions above is *yes*, the affected research team member(s) must complete and submit the form, which is accessible online at http://coi.unc.edu. List name(s) of all research team members for whom any answer to the questions above is *yes*:

____

Certification by Principal Investigator: By submitting this IRB application, I (the PI) certify that the information provided above is true and accurate regarding my own circumstances, that I have inquired of every UNC-Chapel Hill employee or trainee who will be engaged in the design, conduct or reporting of results of this project as to the questions set out above, and that I have instructed any such person who has answered "yes" to any of these questions to complete and submit for approval a Conflict of Interest Evaluation Form. I understand that as Principal Investigator I am obligated to ensure that any potential conflicts of interest that exist in relation to my study are reported as required by University policy.

____ ________________

Signature of Principal Investigator Date

Part A.4. Questions Common to All Studies

A.4.1. **Brief Summary**. Provide a *brief* non-technical description of the study, which will be used in IRB documentation as a description of the study. Typical summaries are 50-100 words. *Please reply to each item below, retaining the subheading labels already in place, so that reviewers can readily identify the content.*

Purpose: The purpose of this study is to compare short term responses to live attenuated influenza virus (via Flumist® vaccine) between subjects treated with broccoli sprout homogenates (BSH) vs. subjects treated with placebo. Effects will be studied in cohorts of smokers and nonsmokers.

Participants: Up to 84 healthy individuals, age 18-40, will be ***enrolled*** in order to ***complete*** a total of 68 subjects-34 subjects (17 treatment, 17 placebo) in each of 2 cohorts: smokers (> 0.5 pack/day) vs nonsmokers. This allows for an attrition/drop out/withdrawal rate of approximately 20%.

Procedures (methods): This protocol will be a randomized, placebo controlled prospective phase 1 study. Our hypothesis is that sulforaphane containing nutritional supplements (such as broccoli sprouts), improve innate immune responses to influenza via Nrf2-linked effects on inflammatory and antiviral pathways. Subjects will be seen for an initial screening visit and, if deemed eligible, will be randomly assigned to receive one of two homogenates. Broccoli sprout homogenate will serve as the active treatment arm since it has high levels of sulforaphane (SFN), while the placebo control arm will be alfalfa sprout homogenate with a low level of SFN. Two to four weeks after screening, subjects will return for 5 sequential visits, Monday through Friday. During the Monday though Thursday daily visits they will receive the assigned homogenate. On Tuesday (Day 0), subjects will receive the FDA approved live attenuated influenza virus (LAIV) vaccine. Subjects will also be seen at 7 days (+/- 1 day) and again at 21 days +/- 7 days. Nasal lavage, blood samples and nasal biopsies will performed prior to and after the study interventions.

A.4.2. **Purpose and Rationale**. Provide a summary of the background information, state the research question(s), and tell why the study is needed. If a complete rationale and literature review are in an accompanying grant application or other type of proposal, only provide a brief summary here. If there is no proposal, provide a more extensive rationale and literature review, including references.

Smokers are more susceptible to influenza infection for reasons that are unclear. Recent work in our laboratory suggests that smokers have suppressed IL-6 and antiviral pathway responses to LAIV, suppressed NK cell activation, reduced Nrf2 expression in nasal epithelium, and increased early viral replication. *In vitro* experiments suggest that epithelial antiviral responses are linked to expression of antioxidants via Nrf2 dependent pathways, and these effects can be reversed with agents that promote Nrf2 activity, including sulphoraphane, leading to reduced viral replication. Sulphoraphane (SFN) is an isothiocyanate which has also generated interest recently as a chemopreventive agent in cancer research, and as an antioxidant in inflammation research. Broccoli sprouts are a concentrated source of the potent phase 2 enzyme (antioxidant) potentiator, SFN, in the form of its natural precursor, sulforaphane glucosinolate (SGS™). *In vivo* data indicate that consumption of homogenates from broccoli sprouts leads to enhanced expression of phase 2 (antioxidant) enzymes in nasal respiratory epithelium and nasal lavage fluid cells (epithelium, NK cells, neutrophils). Additional background information can be found in specific aim 3 section of the accompanying grant application.

For this protocol, we are proposing a randomized, placebo controlled prospective phase 1 study comparing short term responses to LAIV between subjects treated with broccoli sprout homogenates (BSH) vs. placebo. Effects will be studied in cohorts of smokers and nonsmokers.

A.4.3. **Subjects.** *You should describe the subject population even if your study does not involve direct interaction (e.g., existing records).* Specify number, gender, ethnicity, race, and age. Specify whether subjects are healthy volunteers or patients. If patients, specify any relevant disease or condition and indicate how potential subjects will be identified. Researchers are reminded that additional approvals may be needed from relevant "gatekeepers" to access subjects (e.g., school principals, facility directors, hospital or healthcare system administrators).

Up to 84 healthy individuals, age 18-40, will be ***enrolled*** in order to ***complete*** a total of 68 subjects- 34 subjects (17 treatment, 17 placebo) in **each** of 2 cohorts: smokers (> 0.5 pack/day) vs nonsmokers. Gender and ethnicity are not anticipated to have any impact on this study's endpoints and will not be considered in recruitment.

A.4.4. **Inclusion/exclusion criteria.** List required characteristics of potential subjects, and those that preclude enrollment or involvement of subjects or their data. Justify exclusion of any group, especially by criteria based on gender, ethnicity, race, or age. If pregnant women are excluded, or if women who become pregnant are withdrawn, specific justification must be provided.

Inclusion criteria

1. Healthy adults, age 18-40 yr who are either nonsmokers OR smokers (> 0.5 pack/day);
2. Willing to avoid corticosteroids and nonsteroidal anti-inflammatory medications for 1 week prior to study entry and again for 1 week prior to all remaining visits;
3. Willing to avoid antioxidant vitamins and cruciferous vegetables as well as juices/drinks with vitamin supplements added for 2 days prior to Day (-1) of the protocol and throughout the subsequent study.

Exclusion criteria

1. Seasonal allergies requiring medications to control (past 12 months);
2. Respiratory infection (cough, sore throat, sinusitis, fever etc) within prior 4 weeks;
3. Current nutritional disorder such as anorexia, bulimia, irritable bowel syndrome, Crohn's disease etc;
4. Pregnancy or nursing;
5. Asthma (other than wheezing occurring only in childhood); immunodeficiency (HIV or other); or any chronic medical condition that, in the opinion of the investigator, would preclude subject participation;
6. Current use of immunosuppressive drugs;
7. History of fainting or feeling severely dizzy with blood draws;
8. History of hypersensitivity, especially anaphylactic reactions, to egg proteins, gentamicin, gelatin, or arginine or with adverse reactions to previous influenza vaccinations;
9. History of Guillain Barre syndrome;
10. Smokers who have abnormal lung function on pulmonary function testing at the time of screening (FVC and $FEV_1 < 80\%$ of that predicted based on subject age, gender, height and race);
11. Subjects who will be unable to avoid contact with immunocompromised individuals for 3 weeks after receiving LAIV vaccine;
12. Receipt of any type of influenza vaccine since August 2009;
13. Diagnosed influenza illness since August 2009;
14. History of intolerance of or aversion to broccoli.

A.4.5. **Full description of the study design, methods and procedures.** Describe the research study. Discuss the study design; study procedures; sequential description of what subjects will be asked to do; assignment of subjects to various arms of the study if applicable; doses; frequency and route of administration of medication and other medical treatment if applicable; how data are to be collected (questionnaire, interview, focus group or specific procedure such as physical examination, venipuncture, etc.). Include information on who will collect data, who will conduct procedures or measurements. Indicate the number and duration of contacts with each subject; outcome measurements; and follow-up procedures. If the study involves medical treatment, distinguish standard care procedures from those that are research. If the study is a clinical trial involving patients as subjects and use of placebo control is involved, provide justification for the use of placebo controls.

Study design: randomized, placebo controlled prospective phase 1 study comparing short term responses to LAIV between subjects treated with broccoli sprout homogenates (BSH) vs. placebo. Effects will be studied in cohorts of smokers and nonsmokers.

Endpoints:

Primary endpoint: IL-6 in NLF after LAIV inoculation (expressed as area under curve for ratio of IL-6 to baseline) in smokers.

Secondary endpoints: FluB RNA in NLF cells; Nrf2 expression in nasal epithelial cells; tolerance of BSH; NLF cytokines, chemokines, types 1/2 IFN, NK cell activation, and %PMN; NBx Nrf2, cytokine, and phase II enzyme mRNA; serum anti-influenza antibodies; PBMC response to influenza antigen (memory T cells). All endpoint in nonsmokers as secondary analysis.

Exploratory endpoints: Nasal biopsy gene array

Schedule of visits:

Prior to the screening visit, subjects will be asked to avoid cruciferous vegetables (list will be provided to them), corticosteroids and nonsteroidal anti-inflammatory medications for 1 week prior to the initial visit and again for 1 week prior to all remaining visits. They will to also be asked to avoid antioxidant vitamins as well as juices/drinks with added vitamin supplements for 2 days prior to enrollment through study completion.

<u>**Baseline screening**</u>: obtain informed consent, review medical history, concomitant medications, vital signs (VS's), urine collection for analysis of cotinine and SFN metabolites for all subjects and pregnancy (Hcg) test for females of child bearing potential (all female subjects unless they are s/p oophorectomy), physical exam by study MD, complete symptom scoring, collect nasal lavage, and nasal biopsy (both nares), buccal swab collection and venipuncture (up to 75 cc's) will be performed for HIV test, baseline SFN level, immune mediators and SFN-induced gene expression changes. Food diaries will be provided with instructions for subjects at this visit

After successful completion of the screening, the subject will be randomized to receive broccoli sprout homogenates or alfalfa sprout homogenates during the next 4 study visits. Subjects will return 2-4 weeks after screening for 5 daily sequential visits (Monday through Friday).

Monday (Day -1): VS's, review of concomitant medications, urine collection for analysis of cotinine and SFN metabolites for all subjects and Hcg for females of child bearing

potential, nasal lavage, observed ingestion of initial dose and continue food diaries started 3 days prior.

Tuesday (Day 0): VS's, symptom/adverse event evaluation, review of concomitant medications, urine collection for analysis of cotinine and SFN metabolites, observed ingestion of 2nd dose, NL, administration of LAIV, continue food diaries.

Wednesday (Day 1): VS's, symptom/adverse event evaluation, review of concomitant medications, urine collection for analysis of cotinine and SFN metabolites, observed ingestion of 3rd dose, NL and continue food diaries.

Thursday (Day 2): VS's, symptom/adverse event evaluation, review of concomitant medications, urine collection for analysis of cotinine and SFN metabolites, observed ingestion of 4th dose, nasal biopsy (left nare), NL and continue food diaries.

Friday (Day 3): VS's, symptom/adverse event evaluation, review of concomitant medications, urine collection for analysis of cotinine and SFN metabolites,venipuncture (up to 10 cc's for SFN level), NL, continue food diaries.

Day 7 (+/- 1 day): VS's, symptom/adverse event evaluation, review of concomitant medications, urine collection for analysis of cotinine and SFN metabolites, nasal biopsy (right nare), NL, continue food diaries.

Day 21 (+/- 1 week): VS's, symptom/adverse event evaluation, review of concomitant medications urine collection for analysis of cotinine and SFN metabolites, NL, venipuncture (up to 45 cc's) for immune mediators as well as SFN-induced gene expression changes and anti-influenza antibody level, collect completed food diary

Protocol:

	**Screen**	**-1**	**0**	**1**	**2**	**3**	**7**	**21**
Obtain informed consent and medical hx	x							
Physical exam	x							
Buccal swab	x							
Urine Hcg (women child bearing potential)	x	x						
Urine for cotinine/SFN metabolites	x	x	x	x	x	x	x	x
Pulmonary function test/spirometry (smokers only)	x							
Nasal lavage	x	x	x	x	x	x	x	x
Nasal biopsy	x				x		x	
Blood draw (for any of those below)	x					x		x
Serum SFN level	x					x		
T cell resp + anti flu Ab	x							x
HIV	x							
LAIV (Flumist)			x					
BSH/placebo		x	x	x	x			
Nasal symptoms survey	x		x	x	x	x	x	x
GI symptoms survey	x		x	x	x	x	x	x
Food diary		x	x	x	x	x	x	x

Procedure descriptions:

Broccoli sprout homogenate (BSH) and placebo (alfalfa sprout) homogenate preparation**:**

Two hundred grams of BSH is equivalent to approximately 111 grams fresh sprouts (about one 4-oz package). Broccosprouts® (Brassica Protection Products LLC) will be used and are commercially available. The homogenate will be prepared by the CTRC Nutrition Research and Biometabolism Team according to a standardized formula[1]. The sprouts will be homogenized with water using a ratio of 1:1.2 in a clean blender. The homogenate will then be frozen in aliquots at -20 degrees C. The same weight (200grams) of alfalfa sprouts will be prepared in an identical manner for the placebo treatment.

Inoculation of LAIV (FluMist®):

LAIV will be purchased from distributors for MedImmune, Inc. and will be dispensed by the UNC Investigational Drug Service. The nasal spray vaccine will be administered according to the manufacturer's recommendations.

Nasal lavage:

This will be performed according to a method we have previously described (Noah 2000) by repetitive spraying of sterile normal saline irrigation solution (5 ml total) into the nostril, followed by voluntary expelling of fluid by the subject into a specimen collection cup. Both nostrils are lavaged in this way and the resulting nasal lavage fluid (NLF) from both sides is combined. A total cell count is performed using a hemocytometer, and then cytocentrifuge slides are prepared, fixed, and stained using modified Wright stain to enable differential cell count determination. Differential cell counts are made by microscopic evaluation of 200 consecutive cells at high magnification. The remainder of the NLF is centrifuged at 500g x 7 minutes to remove cells and debris, and the cell-free supernatant is stored in aliquots at -80 C until used in mediator assays. Total RNA will be isolated from the cell pellets and analyzed for the expression of antioxidant, inflammatory, and immune genes.

Venipuncture:

Up to 130 ml's will be drawn over the course of the study for SFN levels, immune mediators and peripheral blood monocytic cells for SFN-induced gene expression changes, and the coded samples will be analyzed at CEMALB and at an outside lab. In comparison, at the time of a Red Cross blood donation, approximately 500 cc's are drawn. One tube of the blood drawn at the initial visit will be sent to LabCorp in Burlington, NC for HIV testing since LAIV is a relative contraindication for use in immunocompromised individuals. The only identifier on the tube sent for HIV analysis will be study ID number and, specifically, no date of birth or even gender will be included.

Buccal swab collection:

The inside of the cheeks will be gently brushed using a toothbrush followed by rubbing the region with a cotton tipped swab. DNA from the swab will be analyzed for genes related to antioxidant, inflammatory, and immune responses.

Nasal epithelial scrape/biopsy**:**

To biopsy nasal epithelial cells, cells will be scraped from the inferior surface of the middle turbinates on each side of the nose, using a sterile plastic curette (Rhinoprobe) or a sterile

cytology brush. Specimens will be placed into cold (4^oC) cell culture medium (DMEM with penicillin and streptomycin) for transport to the laboratory. After determination of cell differential (by morphology on Wright stain) and viability, specimens having > 85% viable epithelial cells will be divided into an aliquot of cells to be used for immediate processing for RT-PCR or flow cytometry.

Spirometry or pulmonary function testing (PFT) at screening in smokers only:
This test measures the volume of air that can be exhaled and the rate of airflow during exhalation after a maximal inhalation. Subjects will inhale as deeply as possible, then exhale as rapidly and completely as possible into the spirometer. Measurements obtained from each maneuver include the forced vital capacity (FVC), the forced expiratory volume in the first second (FEV1), the maximal mid-expiratory flow rate (FEF 25-75%) and the peak flow (PF). The largest FVC and FEV1, from at least 3 acceptable trials will be selected for analysis and smokers will be required to have an FVC and FEV1 $\geq$ 80 % of the predicted value for an individual of his/her gender, age, race and height.

At the time of study enrollment subjects will be asked if they are willing to have left over coded samples saved in the UNC CEMALB Repository (UNC IRB approved, IRB # 05-2528) for future yet-to-be designated research purposes. It will be made clear to subjects that they need not consent to storage of excess samples in order to participate in the study. If they are willing, they will be asked to sign the consent form for storage in the CEMALB repository.

A.4.6. **Benefits to subjects and/or society.** Describe any potential for direct benefit to individual subjects, as well as the benefit to society based on scientific knowledge to be gained; these should be clearly distinguished. Consider the nature, magnitude, and likelihood of any direct benefit to subjects. If there is no direct benefit to the individual subject, say so here and in the consent form (if there is a consent form). Do not list monetary payment or other compensation as a benefit.

Individual subjects will benefit by receiving an FDA-approved vaccine against influenza, which may help them avoid significant illness due to influenza virus. Society may benefit by information gained regarding the antioxidant effect of SFN on nasal cells.

A.4.7. **Full description of risks and measures to minimize risks.** Include risk of psychosocial harm (e.g., emotional distress, embarrassment, breach of confidentiality), economic harm (e.g., loss of employment or insurability, loss of professional standing or reputation, loss of standing within the community) and legal jeopardy (e.g., disclosure of illegal activity or negligence), as well as known side effects of study medication, if applicable, and risk of pain and physical injury. Describe what will be done to minimize these risks. Describe procedures for follow-up, when necessary, such as when subjects are found to be in need of medical or psychological referral. If there is no direct interaction with subjects, and risk is limited to breach of confidentiality (e.g., for existing data), state this.

Ingestion of sprout homogenates
In a recently published study[1], 5 of 60 subjects who received broccoli sprouts homogenates, similar to the ones we propose using, experienced "mild" and transient side effects of large bowel movement, nasal congestion, soft stools, increased urination, or flatulence. No serious adverse events were reported. In order to minimize food safety risk associated with Broccosprouts®, the manufacturer, Brassica, follows FDA suggested protocols and requires all

seed to be washed and cleaned with 20,000 ppm calcium hypochlorite prior to sprouting. The plants are grown using only light and water and are then tested for SFN content. The sprouts will be handled by the CTRC kitchen personnel using established food safety procedures for handling raw vegetables and will be immediately processed after they are rinsed.

Risks of LAIV.

Mild flu-like symptoms which may occur in the 7 days following LAIV include cough, runny nose, sore throat, chills, muscle aches, nasal congestion/sinusitis, and tiredness or weakness. Subjects will be warned of these potential side effects and will be allowed to take Tylenol for symptoms. In children under age 5 years, there appears to be a significant increased risk for *asthma symptoms* following LAIV, but this was not seen for older children or adults. Nevertheless, asthma history will be an exclusion to participation in the study. In order to exclude subjects with *COPD*, smokers will be asked to perform spirometry at the screening visit and must demonstrate normal pulmonary function with an FVC and FEV_1 of $\geq$ 80% of predicted at screening in order to continue in the study.

Transmission of virus to immunocompromised individuals can occur for up to 3 weeks after receiving vaccine. Risk for this is estimated at 0.5-2.5% based on studies of transmission in group settings. Current FDA approved prescribing information for Flumist does not prohibit its use in individuals exposed to immunocompromised individuals, but for this study we will minimize this low risk by excluding from the study any individuals who are known to have contact with immunocompromised people, and by advising participants to avoid contact with such individuals for 3 weeks after receiving

Nasal lavage

There are no risks associated with nasal lavage.

Venipuncture

Risks related to venipuncture include discomfort and bruising. Hematoma will be minimized by using experienced phlebotomists and applying pressure for several minutes after venipuncture. The possibility of bruising will be minimized by using experienced phlebotomists and applying pressure for several minutes after venipuncture. A history of fainting or feeling severely dizzy with blood draws will exclude individuals from the study.

Buccal swab collection

The gentle rubbing of the toothbrush or swab may cause minor irritation on your inner cheeks if you have a pre-existing sore. This area can be avoided during the swabbing procedure if you let the staff know the location.

Nasal epithelial scrape/biopsy:

There is a risk of mild bleeding from the nose. This will be treated if it occurs by pressure for 5-10 minutes. Nasal biopsy also is transiently painful.

A.4.8. **Data monitoring and analysis.** Tell how the qualitative and/or quantitative data will be analyzed. Explain how the sample size is sufficient to achieve the study aims. This might include a formal power calculation or explanation of why a small sample is sufficient (e.g., qualitative research, pilot studies). Describe the provisions for monitoring the data to ensure the safety of participants. These plans could range from the investigator monitoring subject data for any safety concerns to a sponsor-based DSMB, depending on the study.

Sample size calculation.
Using data from our previous study in smokers (Noah TL. et al. *Env Health Perspect* 2010 in press) showing mean 13.8 for AUC $_{IL\text{-}6\ ratio}$ (defined below) and assuming a common SD = 10.8 for both placebo and treatment groups, we calculated the power for one-sided t-test of the hypothesis

H_0: $\mu_{trt} \le \Delta$ versus H_1: $\mu_{trt} > \Delta$

We considered a range of treatment effects from 125% to 300% of placebo for the primary endpoint. The minimum sample size required to detect a treatment effect (of 150% of placebo was 17 subjects per group. We anticipate the power will be greater than 80% for our stated hypothesis.

Provisions for monitoring data to ensure safety of participants.
Individuals with a current nutritional disorder such as anorexia, bulimia, irritable bowel syndrome, Crohn's disease etc will be excluded from participation. In addition, individuals who are immunocompromised will be excluded, and HIV testing will be performed at the screening visit. All subjects will also under go a physical exam performed by a study MD at the screening visit.

Subjects will be queried at each visit regarding any adverse GI or other symptoms related to ingestion of the broccoli or alfalfa sprout supplements. They will also be queried regarding any symptoms possible related to the LAIV vaccine such as nasal congestion, rhinnorhea, sore throat etc. Vital signs will be assessed at all visits and subjects will keep food diaries during the study. Steps taken to minimize food safety risk associated with ingestion of the sprout homogenates have been discussed in section A.4.7 above.

Reporting of Unanticipated Events
All unanticipated events that are related (or possibly related) to study participation and involve risks to subjects or others will be reported by the Principal Investigator to the IRB. Grade 4-5 events will be reported immediately to the Principal Investigator (if not present during the procedures), who will report the events to the IRB and NIH sponsor within 24-48 hr. Grade 4 will automatically remove subjects from further participation. Grade 4 or 5 events will automatically place study enrollment on hold and trigger automatic IRB review. (Additional details provided in the CTRC addendum).

Stopping rules

- Nose bleed requiring intervention
- Fainting with blood draw
- Vomiting
- Symptoms of a respiratory infection or probable influenza (as determined by the subject's primary health care provider or in the opinion of the study MD) between the time of the screening visit and Day-1 (pre LAIV day) or day 0 (day for planned administration of LAIV vaccine)

Aggregate Analysis of Adverse Events
The principal investigator, co-investigators, and study staff will review the safety after each subject in the event of any unanticipated events. Additionally, the Principal Investigator will review safety data after each subject and overall protocol safety data when 50% of planned subject enrollment has completed the study.

Data safety monitoring
As minor risks only are involved, no DSMB will be used. The study will be monitored internally by the investigators. At least 10% of individual case report forms and source documents will be reviewed to confirm that all recorded data is verifiable, accurate and complete.

A.4.9. **Will you collect or receive any of the following identifiers?** Does not apply to consent forms.

__ No **_X_** Yes *If yes, check all that apply*:

a. **_X_**Names
b. **_X_**Telephone numbers
c. **_X_**Any elements of dates (other than year) for dates directly related to an individual, including birth date, admission date, discharge date, date of death. For ages over 89: all elements of dates (including year) indicative of such age, except that such ages and elements may be aggregated into a single category of age 90 and older
d. **_X_**Any geographic subdivisions smaller than a State, including street address, city, county, precinct, zip code and their equivalent geocodes, except for the initial three digits of a zip code
e. __ Fax numbers
f. **_X_**Electronic mail addresses
g. **_x_**Social security numbers
h. **_X_**Medical record numbers
i. __ Health plan beneficiary numbers
j. __ Account numbers
k. __ Certificate/license numbers
l. __ Vehicle identifiers and serial numbers (VIN), including license plate numbers
m. __ Device identifiers and serial numbers (e.g., implanted medical device)
n. __ Web universal resource locators (URLs)
o. __ Internet protocol (IP) address numbers
p. __ Biometric identifiers, including finger and voice prints
q. __ Full face photographic images and any comparable images
r. __ Any other unique identifying number, code, or characteristic, other than dummy identifiers that are not derived from actual identifiers and for which the re-identification key is maintained by the health care provider and not disclosed to the researcher

A.4.10. **Identifiers in research data**. Are the identifiers in A.4.9 above linked or maintained with the research data?

_X_ yes __ no

Case report forms (CRF's) will contain identifiers as well as vital signs and subjects' description of any symptoms related to ingestion of BSH or placebo sprout homogenate. Samples will be labeled only with study ID, and sample results will NOT be maintained in the CRF's containing the identifying information with the exception of HIV test results (sent to LabCorp with a coded ID only) as this is being performed for safety reasons only and will not be part of the sample analyses related to study endpoints.

A.4.11. **Confidentiality of the data**. Describe procedures for maintaining confidentiality of the data you will collect or will receive. Describe how you will protect the data from access by those not authorized. How will data be transmitted among research personnel? Where relevant, discuss the potential for deductive disclosure (i.e., directly identifying subjects from a combination of indirect IDs).

Risk for breach of confidentiality will be minimized by encoding samples with access to subject's identity limited to the investigators. CRF's/worksheets generated as part of these

studies will be kept in a locked room, and the databases will be accessible only to investigators and project personnel as listed on page 1 of this application. Subjects' names will not be used in any publications. Research data (sample analysis results) will be identified only by study identification numbers. The blood sample sent to LabCorp for HIV testing will have only the coded study ID (ie, no DOB, gender etc will be indicated on the tube or lab request form). These study ID's will be used to maintain relationships in the data between the various tables. This study will conform to HIPAA guidelines for clinical research.

A.4.12. **Data sharing.** With whom will *identifiable* (contains any of the 18 identifiers listed in question A.4.9 above) data be shared outside the immediate research team? For each, explain confidentiality measures. Include data use agreements, if any.

_X_ No one
__ Coordinating Center**:**
__ Statisticians**:**
__ Consultants**:**
__ Other researchers**:**
__ Registries**:**
__ Sponsors:
__ External labs for additional testing:
__ Journals:
__ Publicly available dataset:
__ Other:

A.4.13. **Data security for storage and transmission**. Please check all that apply.

For electronic data stored on a desk top computer:
_X Secure network _X_ Password access __ Data encryption _X_ Password protected file(s)
__ Other comparable safeguard (describe):

For portable computing devices/external storage devices (e.g. laptop computer, PDA, CDs, memory sticks):
__ Power-on password __ Automatic log-off __ Data encryption __ Password protected file(s)
__ Other comparable safeguard (describe):

For hardcopy data (including human biological specimens, CDs, tapes, etc.):
__ Data de-identified by research team (stripped of the 18 identifiers listed in question A.4.9 above)
_X Locked suite or office __ Locked cabinet
_X_ Data coded by research team with a master list secured and kept separately
__ Other (describe):

A.4.14. **Post-study disposition of identifiable data or human biological materials**. Describe your plans for disposition of data or human biological specimens that are identifiable in any way (directly or via indirect codes) once the study has ended. Describe your plan to destroy identifiers, if you will do so.

After study completion, left over coded samples will be transferred to the UNC CEMALB Repository (UNC IRB approved, IRB # 05-2528) for all subjects who, at the time of enrollment in this protocol, consented to have excess samples stored in the Repository. Samples from subjects who declined to have samples stored in the Repository will be destroyed upon completion of study analyses.

Part A.5. The Consent Process and Consent Documentation (including Waivers)

The standard consent process is for all subjects to sign a document containing all the elements of informed consent, as specified in the federal regulations. Some or all of the elements of consent, including signatures, may be altered or waived under certain circumstances.

- If you will obtain consent in any manner, complete **section A.5.1**.
- If you are obtaining consent, but requesting a waiver of the requirement for a signed consent document, complete **section A.5.2**.
- If you are requesting a waiver of any or all of the elements of consent, complete **section A.5.3**.
- If you need to access Protected Health Information (PHI) to identify potential subjects who will then be contacted, you will need a *limited waiver of HIPAA authorization*. This is addressed in section B.2.

You may need to complete more than one section. For example, if you are conducting a phone survey with verbal consent, complete sections A.5.1, A.5.2, and possibly A.5.3.

A.5.1. **Describe the process of obtaining informed consent from subjects**.
Describe who will be obtaining consent (or permission) and from whom. Include discussion, as relevant, any waiting period between the initial consent discussion and obtaining consent, and steps that will be taken to minimize coercion or undue influence. If children will be enrolled as subjects, describe the provisions for obtaining parental permission and assent of the child. If decisionally impaired adults are to be enrolled, describe the provision for obtaining surrogate consent from a legally authorized representative (LAR). If non-English speaking people will be enrolled, explain how consent in the native language will be obtained. Address both written translation of the consent and the availability of oral interpretation. It is expected that the information in the consent document(s) will be communicated to participants or their LAR. *After you have completed this part A.5.1, if you are not requesting a waiver of any type, you are done with Part A.5.; proceed to Part B.*

Potential subjects will be recruited as discussed in section B.1 below. Subjects will contact the Study Coordinator to discuss the protocol, visits, and eligibility. Additionally, when possible, a copy of the approved consent form will be emailed to the subject for review prior to the initial visit (the subject line in the email will indicate only "study info"). If found to be potentially eligible, the subject will be scheduled to come to the CEMALB research facility on Mason Farm Road in Chapel Hill for the screening visit. At that time, the Study Coordinator or one of the investigators will obtain informed consent by direct interview.

A.5.2. **Justification for a waiver of *written* (i.e., signed) consent**. *The default is for subjects to sign a written document that contains all the elements of informed consent.* Under limited circumstances, the requirement for a signed consent form may be waived by the IRB if either of the following is true. *Choose only one:*

NA

a. The only record linking the subject and the research would be the consent document and the principal risk would be potential harm resulting from a breach of confidentiality (e.g., study topic is sensitive so that public knowledge of participation could be damaging). *Participants should be asked whether they want documentation linking them with the research and the participants' wishes will govern whether they sign the form.* Note: This justification cannot be used in FDA-regulated research. __ yes __ no

__ yes __ no

Explain.

b. The research presents no more than minimal risk of harm to subjects and involves no procedures for which written consent is normally required outside of the research context (e.g., phone survey).
Explain.

If you checked "yes" to either (and you are not requesting a waiver in section A.5.3) consent must be obtained orally, by delivering a fact sheet, through an online consent form, or be incorporated into the survey itself. Include a copy of the consent script, fact sheet, online consent form, or incorporated document.

→ If you have justified a waiver of written (signed) consent (A.5.2), you should complete A.5.3 *only* if your consent process will not include all the other elements of consent.

A.5.3. **Justification for a full or partial waiver of consent.** *The default is for subjects to give informed consent.* A waiver might be requested for research involving only existing data or human biological specimens (see also Part C). More rarely, it might be requested when the research design requires withholding some study details at the outset (e.g., behavioral research involving deception). In limited circumstances, parental permission may be waived. This section should also be completed for a waiver of HIPAA authorization if research involves Protected Health Information (PHI) subject to HIPAA regulation, such as patient records.

NA

__ Requesting **waiver of some elements** (specify; see SOP 28 on the IRB web site):
__ Requesting **waiver of consent entirely**
If you check either of the boxes above, answer items a-f.. To justify a full waiver of the requirement for informed consent, you must be able to answer "yes" (or "not applicable" for question c) to items a-f. **Insert brief explanations that support your answers.**

a. Will the research involve no greater than minimal risk to subjects or to their privacy? __ yes __ no
Explain.

b. Is it true that the waiver will *not* adversely affect the rights and welfare of subjects? *(Consider the right of privacy and possible risk of breach of confidentiality in light of the information you wish to gather.)* __ yes __ no
Explain.

c. When applicable to your study, do you have plans to provide subjects with pertinent information after their participation is over? *(e.g., Will you provide details withheld during consent, or tell subjects if you found information with direct clinical relevance? This may be an uncommon scenario.)* __ yes __ not applicable
Explain.

d. Would the research be impracticable without the waiver? *(If you checked "yes," explain how the requirement to obtain consent would make the research impracticable, e.g., are most of the subjects lost to follow-up or deceased?).* __ yes __ no
Explain.

e. Is the risk to privacy reasonable in relation to benefits to be gained or the __ yes __ no

importance of the knowledge to be gained?
Explain.

If you are accessing patient records for this research, you must also be able to answer "yes" to item f to justify a waiver of HIPAA authorization from the subjects.

f. Would the research be impracticable if you could not record (or use) Protected Health Information (PHI)? *(If you checked "yes," explain how* not *recording or using PHI would make the research impracticable).* __ yes __ no
Explain.

Part B. Questions for Studies that Involve Direct Interaction with Human Subjects

→ ***If this does not apply to your study, do not submit this section.***

B.1. **Methods of recruiting.** Describe how and where subjects will be identified and recruited. Indicate who will do the recruiting, and tell how subjects will be contacted. Describe efforts to ensure equal access to participation among women and minorities. Describe how you will protect the privacy of potential subjects during recruitment. *For prospective subjects whose status (e.g., as patient or client), condition, or contact information is not publicly available (e.g., from a phone book or public web site), the initial contact should be made with legitimate knowledge of the subjects' circumstances. Ideally, the individual with such knowledge should seek prospective subjects' permission to release names to the PI for recruitment. Alternatively, the knowledgeable individual could provide information about the study, including contact information for the investigator, so that interested prospective subjects can contact the investigator.* Provide the IRB with a copy of any document or script that will be used to obtain the patients' permission for release of names or to introduce the study. Check with the IRB for further guidance.

Potential subjects will be recruited using local advertisements, CEMALB website posting, and informational emails sent to the University staff and students. Subjects responding to ads will be asked to contact the Study Coordinator. Participants will also be recruited from our database of subjects who have completed the CEMALB screening protocol (IRB #98-CEMLB-293) and who have agreed to be contacted in the event they are eligible for specific studies. Phone conversations with potential participants will take place in a private office. If the volunteer agrees to participation, he/she will be scheduled to come to the CEMALB research facility in the US EPA Human Studies Facility on Mason Farm Road in Chapel Hill for the initial study visit.

B.2. **Protected Health Information (PHI).** If you need to access Protected Health Information (PHI) to identify potential subjects who will then be contacted, you will need a *limited waiver of HIPAA authorization*. If this applies to your study, please provide the following information and complete Section C.

NA

a. Under this limited waiver, you are allowed to access and use only the minimum amount of PHI necessary to review eligibility criteria and contact potential subjects. What information are you planning to collect for this purpose?

b. How will confidentiality/privacy be protected prior to ascertaining desire to participate?

c. When and how will you destroy the contact information if an individual declines participation?

B.3. **Duration of entire study and duration of an individual subject's participation, including follow-up evaluation if applicable.** Include the number of required contacts and approximate duration of each contact.

Duration of the entire study: 2 years. Duration of individual subject's participation: about 1½ -2 months (screening visit followed 2-4 weeks later by entry into study for 21 days +/- 1 week). The screening visit will be up to 2 hours in duration and subsequent visits will be approximately 1 hour.

B.4. **Where will the subjects be studied?** Describe locations where subjects will be studied, both on and off the UNC-CH campus.

Subjects will be studied a the clinical research facility of the UNC Center for Environmental Medicine, Asthma and Lung Biology (CEMALB), 104 Mason Farm Rd., Chapel Hill, NC.

B.5. **Privacy.** Describe procedures that will ensure privacy of the subjects in this study. Examples include the setting for interviews, phone conversations, or physical examinations; communication methods or mailed materials (e.g., mailings should not indicate disease status or focus of study on the envelope).

All interviews will be carried out in private examination rooms or private offices in the CEMALB research facility. All phone conversations will also be carried out in private offices. All physical examinations will be carried out in a private examination room.

B.6. **Inducements for participation.** Describe all inducements to participate, monetary or non-monetary. If monetary, specify the amount and schedule for payments and if/how this will be prorated if the subject withdraws (or is withdrawn) from the study prior to completing it. For compensation in foreign currency, provide a US$ equivalent. Provide evidence that the amount is not coercive (e.g., describe purchasing power for foreign countries). Be aware that payment over a certain amount may require the collection of the subjects' Social Security Numbers. If a subject is paid more than $200.00 per year, collection of subjects' Social Security Number is required (University policy—see SSN Guidance) using the Social Security Number collection consent addendum found under forms on the IRB website (look for Study Subject Reimbursement Form).

Screening visit	$70
Day-1	$30
Day 0	$30
Day 1	$30
Day 2	$30
Day 3	$70
Day 7	$55
Day 21	$45
Completion bonus (attend all visits and return food diary	$25
	$385

Thus an individual subject who completes all procedures will be paid $385 and will be provided with vouchers for free parking. Subjects who do not complete the study will be paid for completed visits.

B.7. **Costs to be borne by subjects.** Include child care, travel, parking, clinic fees, diagnostic and laboratory studies, drugs, devices, all professional fees, etc. If there are no costs to subjects other than their time to participate, indicate this.

No costs will be borne by the subject, other than time to participate. Parking fees will be paid by the study.

Reference:

1. Riedl MA, Saxon A, Diaz-Sanchez D. Oral sulforaphane increases Phase II antioxidant enzymes in the human upper airway. *Clin Immunol* 2009; 130:244-251
